# Spelling impairments in Spanish dyslexic adults

**DOI:** 10.3389/fpsyg.2015.00466

**Published:** 2015-04-20

**Authors:** Olivia Afonso, Paz Suárez-Coalla, Fernando Cuetos

**Affiliations:** ^1^Departamento de Psicología Cognitiva, Social y Organizacional, Universidad de La LagunaSanta Cruz de Tenerife, Spain; ^2^Departamento de Psicología, Universidad de OviedoOviedo, Spain

**Keywords:** developmental dyslexia, spelling to dictation, copying, word frequency, P-O consistency, word length, writing durations

## Abstract

Spelling deficits have repeatedly been observed in children with dyslexia. However, the few studies addressing this issue in dyslexic adults have reported contradictory results. We investigated whether Spanish dyslexics show spelling deficits in adulthood and which components of the writing production process might be impaired in developmental dyslexia. In order to evaluate the involvement of the lexical and the sublexical routes of spelling as well as the graphemic buffer, lexical frequency, phonology-to-orthography consistency and word length were manipulated in two writing tasks: a direct copy transcoding task and a spelling-to-dictation task. Results revealed that adults with dyslexia produced longer written latencies, inter-letter intervals, writing durations and more errors than their peers without dyslexia. Moreover, the dyslexics were more affected by lexical frequency and word length than the controls, but both groups showed a similar effect of P-O consistency. Written latencies also revealed that while the dyslexics initiated the response later in the direct copy transcoding task than in the spelling-to-dictation task, the controls showed the opposite pattern. However, the dyslexics were slower than the controls in both tasks. Results were consistent with the hypothesis that spelling difficulties are present in adults with dyslexia, at least in a language with a transparent orthography such as Spanish. These difficulties seem to be associated with a deficit affecting both lexical processing and the ability to maintain information about the serial order of the letters in a word. However, the dyslexic group did not differ from the control group in the application of the P-O conversion procedures. The spelling impairment would be in addition to the reading deficit, leading to poorer performance in direct copy transcoding compared to spelling-to-dictation.

## Introduction

It is well-documented that spelling deficits are present in most cases of developmental dyslexia, and it seems that these problems may persist into adulthood (Lefly and Pennington, 1991; Di Betta and Romani, 2006; Tops et al., 2012; Bogdanowicz et al., 2014). This impairment is likely to have negative consequences on academic and professional development, since dyslexics produce poor texts with multiple misspellings. Thus, writing difficulties are one of most frequent complaints among adults (Holmes and Castles, 2001; Di Betta and Romani, 2006). However, only a few studies have focused on the nature of the spelling impairment in dyslexia and its development across the life span, so many questions remain unanswered. This is in part due to the fact that research about the writing production process is relatively recent.

According to dual-process theories, spelling may be achieved through at least two different processing routes. The *sublexical* or *assembled route* makes use of knowledge about phonology-to-orthography (P-O) correspondences existing in the language, and provides phonologically plausible spellings for non-words or low-frequency words (Caramazza, 1988; Tainturier and Rapp, 2001). The so-called *lexical route* gives access to the spelling of whole-words from long-term memory, and thus would be used when spelling familiar words. The existence of both routes is almost undisputed, even in particularly shallow orthographies such as Spanish or Italian, in which spelling could be accomplished by resorting merely to the P-O conversion procedures (Valle, 1989; Cuetos, 1991, 1993; Barry and De Bastiani, 1997; Cuetos and Labos, 2001). Moreover, the independence of these routes is supported by the fact that they can be selectively damaged (Beauvois and Dérouesné, 1981; Shallice, 1988; Tainturier and Rapp, 2001). In *surface dysgraphia*, the lexical route is affected, so errors appear in words with non-predictable spellings (inconsistent and irregular words) as a result of the overreliance on the phoneme-to-grapheme conversion procedures. In the case of *phonological dysgraphia*, word spelling is spared, but non-word spelling is affected due to difficulties in correctly applying the P-O correspondences.

Growing evidence suggests that both routes interact during the spelling process. Specifically, some authors have proposed that lexical and sublexical information could integrate at a grapheme level (Tainturier and Rapp, 2001; Rapp et al., 2002; Bosse et al., 2003; Afonso and Álvarez, 2011). Both processes (lexical and sublexical) would “vote for” a candidate graphemic element, so when both systems produce the same output (the same grapheme is activated) the selection would be reinforced. As a result, sublexical phonological information would influence the writing of well-known words at the level of grapheme selection. Most of the evidence suggesting interaction of lexical and sublexical processes has revealed the influence of lexical knowledge on the sublexical route (Campbell, 1983; Barry and Seymour, 1988; Cuetos, 1991, 1993; Tainturier et al., 2000). However, some studies support that P-O correspondences also have an impact during word spelling (Bonin et al., 2001b; Folk and Rapp, 2004; Zhang and Damian, 2010; Afonso and Álvarez, 2011; Afonso et al., 2014).

Regardless of the route followed to access the orthographic representation, this representation has been claimed to be held in a short-term memory store, the so-called *graphemic buffer*, in which the abstract graphemic units are kept for subsequent production (Cuetos, 1991; Tainturier and Rapp, 2003). The orthographic sequences computed by lexical or sublexical processes would be maintained by the buffer during the time needed for the sequential assignment of format-specific information depending on the output modality (for example, letter name in oral spelling or letter shape in handwriting). Patients with *graphemic buffer deficits* (Caramazza et al., 1987; Buchwald and Rapp, 2004; Tainturier and Rapp, 2004) make errors affecting individual graphemes (substitutions, deletions, additions, transpositions), and they show a significant effect of word length. Moreover, they perform comparably across modality of input (copying, spelling-to-dictation, written picture naming), across modality of output (typing, handwriting, oral spelling), and across type of stimulus (regular and irregular word, non-words). This pattern of errors seems to reflect a difficulty in maintaining serially-ordered abstract letter representations.

Some authors have claimed that the spelling impairment observed in dyslexia might be associated with phonological factors. Traditionally, phonological awareness has been associated with the spelling difficulties experienced by dyslexics (Goswami and Bryant, 1990; Caravolas et al., 2001; Shaywitz and Shaywitz, 2005; Friend and Olson, 2010). In contrast, some evidence suggests that dysgraphia in developmental dyslexia may emerge as a consequence of poor orthographic lexical representations (Goulandris and Snowling, 1991; Hanley et al., 1992; Angelelli et al., 2004; Di Betta and Romani, 2006).

One reason that might explain this lack of agreement in the literature is the fact that the studies addressing this issue have been conducted with samples of very different ages. It has been observed that spelling deficits are different in children and adults with dyslexia. In fact, different writing impairments have been described for children from different school grades. In a study with Italian dyslexic children attending Grades 3 and 5 (Angelelli et al., 2010), younger dyslexic children committed all type of errors while older children primarily made phonologically plausible errors. In another study, Di Betta and Romani (2006) asked dyslexic adults to learn spoken and written pseudowords and Dutch words in association to pictures. They found that the learning curve exhibited by adults with developmental dyslexia in reading and writing tasks was impaired from the first trial and that it remained impaired. Correlational analyses revealed that this difficulty in lexical learning was independent of the phonological deficits usually observed in dyslexic children. Altogether, these results are in line with the claim that spelling impairments in dyslexia may be a function of age (Wimmer, 1996; Bishop, 1997; Snowling and Nation, 1997; Landerl and Wimmer, 2000; Angelelli et al., 2010; Tops et al., 2012).

Finally, it has also been proposed that the core deficit in developmental dyslexia might affect the short-term memory system (Ramus and Szenkovits, 2008; Menghini et al., 2011) or the ability to process and/or access temporal order information (Beneventi et al., 2009; Szmalec et al., 2011). Szmalec et al. (2011) suggested that a difficulty to generate serial-ordered long-term representations from a sequence in short-term memory may be the cause of the spelling difficulties associated with developmental dyslexia. However, some findings have contradicted these hypotheses (Di Betta and Romani, 2006; Tops et al., 2012). In their study, Tops et al. (2012) observed that dyslexics did not make more transposition errors than controls. They interpreted this result as evidence of preservation of letter order information.

In the present study we aimed to shed light on the nature of spelling deficits in adults with developmental dyslexia. Specifically, we investigated how the main components of the writing production system (P-O conversion procedures, orthographic output lexicon, and graphemic buffer) are involved during written word production in both dyslexic and non-dyslexic adults in a language with a transparent orthography such as Spanish. In order to fulfill this objective, we examined the impact of P-O consistency, word frequency and word length in two different writing tasks. These variables are thought to reflect the use of the sublexical route, the lexical route and the graphemic buffer respectively during the spelling process.

Effects of P-O consistency during spelling have been reported in the literature (Ziegler et al., 1996; Bonin et al., 2001b, 2014). Along with effects of P-O regularity (Kandel and Valdois, 2005; Delattre et al., 2006; Roux et al., 2013), P-O consistency effects have been interpreted as evidence of the application of the conversion procedures linking phonemes and graphemes. More errors would be made in inconsistent than in consistent words because in the former case successful spelling cannot be achieved by resorting only to knowledge of the P-O conversion rules. Thus, if the correct orthographic representation is not stored in the lexicon, the sublexical route would be likely to produce an incorrect form. Even when the orthographic word-form is available, P-O consistency effects are expected to be observed in written latencies. As mentioned above, both routes are thought to be involved during the normal spelling process, and the graphemic units will be reinforced when both routes produce the same output. When spelling P-O consistent words, the sublexical route would activate the same graphemes activated by the lexical route; however, when there is a P-O inconsistent segment in the word, the sublexical route would produce more than one potential spelling. Therefore, graphemes corresponding to P-O consistent segments would be strengthened in comparison to P-O inconsistent segments. Regarding word frequency, this variable has not been as thoroughly studied in handwriting research as in, for example, visual word recognition. However, some evidence suggests that orthographic representations are stored in the lexicon weighted according to their frequency, leading to shorter written latencies for high-frequency than for low-frequency words (Bonin et al., 2001a; Bonin and Fayol, 2002). Finally, marked word length effects observed in dysgraphic patients have been linked to the short-term memory system (Tainturier and Caramazza, 1996; Miceli et al., 1997; Tainturier and Rapp, 2003), but this variable has not been systematically studied in non-dysgraphic populations (although see Van der Plaats and Van Galen, 1990). We believe that the study of the effects of word frequency, P-O consistency and word length allow us to identify the component or components of the writing production system that might be deficient in adults with dyslexia.

Recruitment of the graphemic buffer seems to be required during any spelling task. However, it is widely admitted that the impact of the lexical and the sublexical routes during writing depends, among other things, on the type of task being performed (Cuetos, 1991; Bonin et al., 2001b, 2014). For example, Bonin et al. (2001b) observed that the presence of inconsistencies in the P-O mappings affected written latencies during a written picture naming task only when inconsistencies appeared at initial positions. However, during spelling-to-dictation written latencies were affected by the presence of initial, middle and final inconsistencies. The authors concluded that the semantic influence on orthographic encoding when writing words from pictures allowed final inconsistencies to be solved before production. Nevertheless, analyses of errors made by dysgraphic patients have revealed effects of P-O consistency at all positions (Rapp et al., 2002; Tainturier and Rapp, 2003) even in written picture naming (Folk et al., 2002). Thus, it is yet unclear to what extent each particular process influences spelling depending on the demands of the task.

In the present study we manipulated P-O consistency, word frequency and word length and tested the same words in a direct copy transcoding task and in a spelling-to-dictation task with a three-fold aim. First, we attempted to compare the impact of these variables on each task. P-O consistency may have an effect only in the spelling-to-dictation task, since access to the phonological representation is not required to perform the direct copy transcoding task. Second, we sought to establish whether or not the dyslexic group has impaired spelling abilities and how their deficit affects different tasks. For example, dyslexic adults may not show impaired spelling abilities *per se*, but only reading difficulties. Thus, differences between the dyslexic and the non-dyslexic group might reveal only differences in the copying task due to the potential involvement of reading processes, but not in spelling-to-dictation when the input is auditory. However, if the dyslexic group has an independent deficit affecting the writing production system, then differences between groups should be observed in both tasks. Finally, and if the dyslexics show spelling impairments, we aim to determine which specific processes might underlie those difficulties. Effects of P-O consistency, word frequency or word length in the dyslexic group differing from those obtained for the control group may suggest the existence of differences in the use of sublexical information, lexical information or the graphemic buffer respectively.

We collected written latencies, inter-letter interval (ILI) durations and whole-word writing durations from a group of adults with dyslexia and a control group. While written latencies are thought to reflect access to the orthographic representation (central processes), writing and ILI durations are considered to tap into more peripheral processes. However, recent evidence suggests that central processing may also affect writing and ILI durations (Roux et al., 2013). It has been recently observed that sublexical phonological information can produce an effect in ILI durations in the direct copy transcoding task without affecting written latencies (Afonso, 2014; Afonso et al., 2014). These findings may reflect the fact that sublexical phonological information can be used to reinforce the orthographic representations at the grapheme level, producing an effect only when the specific grapheme has to be actually produced. Moreover, besides written latencies (Van Galen et al., 1986), ILI durations should be strikingly affected by word length effects. Graphemic buffer deficits affect the correct retrieval of the individual graphemes, and patients with this type of deficits made fewer errors at initial than at middle positions (Tainturier and Rapp, 2003, 2004; Buchwald and Rapp, 2004). This evidence suggests that the involvement of the buffer is less relevant at the beginning of the written response. The analysis of written latencies and ILI durations in addition to the analysis of errors allows us to obtain a more detailed picture of the writing process, and it also makes it possible to detect more subtle differences between dyslexic and control participants. To our knowledge, this is the first study addressing the spelling abilities of individuals with dyslexia that provides chronometric measures of the written response.

## Materials and methods

### Participants

Twenty dyslexic adults (8 males and 12 females) and 20 normal reading adults (8 males and 12 females) participated in this study. Although in studies testing dyslexic children reading age instead of chronological age is often used for matching the dyslexic and the control groups, chronological age seems to be a more adequate criterion in the case of adult participants. Using reading age should lead to dramatic differences in other important variables, such as chronological age (in some cases it might require even the inclusion of children in the control group) or years of education. For this reason, we chose to match participants across groups by chronological age, in addition to years of education and sex. All the participants were native Spanish speakers and had no known motor or perceptual disorders. Participants were recruited by promoting information about the experiment on the University campus and by publishing this information in the University newspaper and other local publications. The participants with dyslexia were diagnosed based on self-reported history of reading problems in childhood and their performance on reading achievement tests. A battery designed to asses reading, PROLEC-SE (Ramos and Cuetos, 1999), and a battery to assess spelling, PROESC (Cuetos et al., 2002), were administrated to all participants. PROLEC-SE provides scores for word and pseudoword reading. The word reading section includes 40 Spanish words, half of them being high-frequency words and the other half low-frequency words. For each half, 10 words are short words and 10 are long words. Regarding pseudowords, half of them were short and the other half were long. PROESC includes a section for writing 25 words with arbitrary orthography, a section for writing 25 words with non-arbitrary orthography (there is a rule in Spanish determining the spelling of these words) and a section for writing 25 pseudowords. Digit span was also evaluated. Volunteers that had reported reading difficulties in childhood were included in the dyslexic group if they scored more than 1.5 standard deviations below the control group in both the word and pseudoword reading sections of PROLEC-SE. Means, standard deviations and *p*-values for demographic characteristics and scores obtained in the digit span test and in reading and spelling assessment tests are provided in Table 1. None of the dyslexic participants had received systematic treatment for their reading impairment.

**Table 1:** **Means and standard deviations for demographic characteristics, reading and spelling scores of control and dyslexic participants**.

	**Controls**	**Dyslexics**	***p*-value**
Age	35 (12.55)	35 (11.61)	0.991
Education (years)	15.25 (2.24)	15.25 (2.07)	1
**READING**					
**Words**			
Accuracy	39.9/40 (0.31)	38.7/40 (1.30)	<0.001
Speed (s)	23.75 (6.23)	36.25 (10.20)	<0.001
**Pseudowords**			
Accuracy	38.8/40 (2.11)	34.87/40 (2.91)	<0.001
Speed (s)	36.75 (6.88)	62.15 (14.74)	<0.001
**SPELLING**					
**Arbitrary orthography**			
Accuracy	24.2/25 (1.24)	22.85/25 (2.16)	<0.05
**Non-arbitrary orthography**			
Accuracy	24.3/25 (1.08)	21.9/25 (2.47)	<0.001
**Pseudowords**			
Accuracy	22.4/25 (1.73)	20.65/25 (2.68)	<0.05
**DIGIT SPAN**					
Accuracy	11.45 (1.50)	10.45 (1.99)	0.054

### Design

A 2 × 2 × 2 × 2 × 2 design was applied. Group (control vs. dyslexic) was a between-subjects variable, and type of task (direct copy transcoding vs. spelling-to-dictation), P-O consistency (consistent vs. inconsistent), word frequency (high vs. low) and word length (short vs. long) were within-subjects variables.

### Materials

Thirty-two Spanish common nouns were selected as experimental stimuli. Among them, phoneme-to-grapheme consistency (consistent vs. inconsistent), word frequency (high vs. low), and word length (short vs. long) were orthogonally varied. Inconsistent words included one grapheme with at least one alternative spelling. For example, the word *TELEVISIÓN* ([teleβi′sjon], *television*) is inconsistent because it includes the phoneme /β/, which could be spelled *B* instead of *V*. Consistent words only included phonemes with unambiguous spellings (e.g., *TELÉFONO*, *phone*). Across the consistency conditions, words were matched in word frequency, word length (number of letters and syllables), orthographic neighborhood and age of acquisition (AoA). For the lexical frequency manipulation, words with a frequency above 13.5 according to values provided by BuscaPalabras (Davis and Perea, 2005) were considered high-frequency words and those with a frequency below 6.5 were considered low-frequency words. Across the word frequency conditions, words were matched in word length (number of letters and syllables), orthographic neighborhood and AoA. Regarding word length, short words had 4–6 letters and long words had 7–11 letters. In this case, both conditions were matched in word frequency, orthographic neighborhood, and AoA. The full set of experimental stimuli with the values provided by BuscaPalabras (Davis and Perea, 2005) for independent and controlled variables are given in Supplementary Material. For each word, a visual and an auditory stimulus were created for the direct copy transcoding and the spelling-to-dictation task respectively.

### Apparatus

Stimuli presentation and digital recording of the responses were controlled by Ductus (Guinet and Kandel, 2010). The experiment was run on an HP Mini laptop. A WACOM Intuos 5 graphic tablet connected to the computer and an Intuos Pen were used to register the participants' responses. Auditory stimuli were recorded by a female speaker with a Plantronics microphone and edited with Audacity.

### Procedure

The procedure of this experiment was approved by the Ethics Committee of the Department of Psychology of the University of Oviedo. The experiment was conducted individually in a quiet room. For all the participants the spelling-to-dictation task was conducted before the direct copy transcoding task.

In the spelling-to-dictation task, each trial started with the simultaneous presentation of an auditory signal and a 500-ms fixation point. The auditory stimulus was presented 500 ms after the offset of the fixation point. Participants had to write the word in upper case (print handwriting was not enforced) on a lined sheet of paper placed over the digitizer as quickly and as accurately as possible. The ink was removed from the pen, so participants did not see their responses. By doing so, we ensured that potential effects could not be due to visual feedback from previous written outputs. When they finished a response, participants were instructed to hold the pen over the next line of the response sheet, but without making any contact with the paper. Then, the experimenter clicked the left button of the mouse to start a new stimulus.

In the direct copy transcoding task, a trial started with the same auditory signal and fixation point as in the spelling-to-dictation task, and was followed by a 500-ms white screen. Then, the visual stimulus was presented in black lower-case Calibri 60 point font on a white background, and it remained onscreen until the next stimulus was presented. The instructions given to the participants were the same than in the spelling-to-dictation task. Their attention was called to the fact that they had to continue writing the words in upper case, in spite of the fact that they would see the stimulus in lower case. The whole experiment lasted around 15 min.

## Results

Written latencies, durations for the critical ILI (the critical ILI was, in the inconsistent words, the ILI immediately prior to the inconsistent segment, and in their matched consistent words, the ILI located at the same position; for example, *SA*_*BOR* and *DE*_*DAL*), writing durations for the whole word and mean percentages of correct responses were submitted to separate analyses of variance (ANOVAs), with group as a between-subjects variable, and type of task, P-O consistency, word frequency, and word length as within-subjects variables. Only correct responses were included in the analyses. Responses containing misspellings and self-corrections or those in which a recording error occurred were considered errors and removed from the analysis (6.25%). Latencies above and below 2 standard deviations from the mean by participant were also excluded from the analysis (3.98%).

### Written latencies

This variable was measured as the time between the presentation of the stimulus and the occurrence of the first contact of the pen with the digitizer. Table 2 shows the means and standard deviations for written latencies in each condition for both groups. The main effects of group, *F*_(1, 38)_ = 17.11; *p* < 0.001; *MSE* = 5,510,835.22; η^2^_*p*_ = 0.31, consistency, *F*_(1, 38)_ = 4.17; *p* < 0.05; *MSE* = 35,343.02; η^2^_*p*_ = 0.10, word frequency, *F*_(1, 38)_ = 20.90; *p* < 0.001; *MSE* = 143,161.22; η^2^_*p*_ = 0.35 and word length, *F*_(1, 38)_ = 37.49; *p* < 0.001; *MSE* = 429,007.66; η^2^_*p*_ = 0.50, were significant. The dyslexic group took longer than the control group to initiate the written response. As expected, written latencies were longer for inconsistent than for consistent words, for low-frequency words compared to high-frequency words, and for long words compared to short words. The interaction Type of task × Group was significant, *F*_(1, 38)_ = 17.51; *p* < 0.001; *MSE* = 2,040,780.62; η^2^_*p*_ = 0.31. *T*-tests revealed that the controls were significantly slower in the spelling-to-dictation task than in the copying task, *t*_(159)_ = 9.55, *p* < 0.001. In contrast, the dyslexic group produced longer written latencies in the direct copy transcoding task than in the spelling-to-dictation task, *t*_(159)_ = 5.92, *p* < 0.001. The interaction between word frequency and group was significant as well, *F*_(1, 38)_ = 4.97; *p* < 0.05; *MSE* = 34,076.41; η^2^_*p*_ = 0.12. The word frequency effect was larger in the dyslexic group than in the control group, *t*_(159)_ = 2.16, *p* < 0.05. Also significant was the interaction between type of task and word length, *F*_(1, 38)_ = 18.13; *p* < 0.001; *MSE* = 138,121.26; η^2^_*p*_ = 0.32. This interaction revealed that the effect of word length affected written latencies in the copying task more than in the spelling-to-dictation task, *t*_(159)_ = 4.25, *p* < 0.001. The three-way interaction Consistency × Word frequency × Word length was significant, *F*_(1, 38)_ = 4.30; *p* < 0.05; *MSE* = 27,693.91; η^2^_*p*_ = 0.10. *T*-tests showed that for consistent words the word frequency effect significantly affected short words, *t*_(79)_ = 1.98, *p* < 0.05, and long words, *t*_(79)_ = 3.71, *p* < 0.001. However, word frequency had an effect on inconsistent short words, *t*_(79)_ = 5.65, *p* < 0.05, but not on inconsistent long words, *t*_(79)_ = 1.58, *p* = 0.11. The four-way interaction Group × Type of task × Consistency × Word frequency, *F*_(1, 38)_ = 5.25; *p* < 0.05; *MSE* = 34,751.02; η^2^_*p*_ = 0.12, was also significant. Planned-comparisons revealed that in the copying task the group effect was larger for inconsistent low-frequency words than for inconsistent high-frequency words, *t*_(39)_ = 2.45, *p* < 0.05, but differences between groups were similar for consistent words regardless of word frequency, *t* < 1. In spelling-to-dictation the opposite pattern was observed. The difference between the dyslexic and the non-dyslexic group was larger for low-frequency words than for high-frequency words in the consistent condition, *t*_(39)_ = 2.45, *p* < 0.05, but was similar in the inconsistent condition for both high- and low-frequency words, *t*_(39)_ = 1.13, *p* = 0.27.

**Table 2:** **Mean written latencies (in milliseconds) and standard deviations (in parentheses) of control and dyslexic participants**.

		**Controls**				**Dyslexics**			
		**Spelling-to-dictation**		**Direct copy transcoding**		**Spelling-to-dictation**		**Direct copy transcoding**	
		**HF**	**LF**	**HF**	**LF**	**HF**	**LF**	**HF**	**LF**
P-O consistent	Long	902 (133)	922 (164)	803 (168)	871 (151)	944 (145)	1020 (156)	1107 (238)	1170 (298)
	Short	918 (138)	887 (125)	761 (154)	778 (154)	953 (128)	1009 (161)	1018 (207)	1063 (263)
P-O inconsistent	Long	937 (140)	934 (114)	820 (149)	839 (177)	1035 (131)	1020 (159)	1143 (291)	1191 (306)
	Short	893 (119)	941 (124)	784 (132)	769 (156)	950 (94)	985 (192)	1037 (208)	1085 (280)

### Critical inter-letter interval durations

An ILI was defined as the time between the last pen lift produced in a letter and the first pen down produced in the following letter. The ILI before the inconsistent segment and the ILI in the same position in the matched consistent words were considered the critical ILI. Table 3 shows the means and standard deviations for critical ILI durations in each condition for both groups. The main effect of group was significant, *F*_(1, 38)_ = 9.05; *p* < 0.01; *MSE* = 383,817.08; η^2^_*p*_ = 0.19. The dyslexics produced longer ILIs than the controls. The main effect of type of task, *F*_(1, 38)_ = 5.79; *p* < 0.05; *MSE* = 51,355.14; η^2^_*p*_ = 0.13, the main effect of consistency, *F*_(1, 38)_ = 4.57; *p* < 0.05; *MSE* = 7819; η^2^_*p*_ = 0.10, and the main effect of word length, *F*_(1, 38)_ = 742.13; *p* < 0.001; *MSE* = 8,045,865.75; η^2^_*p*_ = 0.95, were significant. ILIs were longer in the spelling-to-dictation task than in the copying task, in the inconsistent than in the consistent condition and for long compared to short words. The two-way interaction Word length x Group was significant, *F*_(1, 38)_ = 7.08; *p* < 0.05; *MSE* = 76,803.31; η^2^_*p*_ = 0.16. The effect of length was larger in the dyslexic group than in the control group, *t*_(159)_ = 4.46, *p* < 0.001. Also significant was the interaction between type of task and consistency, *F*_(1, 38)_ = 19.86; *p* < 0.001; *MSE* = 38,703.95; η^2^_*p*_ = 0.34. The effect of consistency was significant in the spelling-to-dictation task, *t*_(159)_ = 3.8; *p* < 0.001, but not in the copying task, *t* < 1. The three-way interaction Type of task × Consistency × Word length was significant, *F*_(1, 38)_ = 8.51; *p* < 0.01; *MSE* = 19,217.26; η^2^_*p*_ = 0.18. *T*-tests revealed that consistency had no effect either in short or long words during copying. However, in spelling-to-dictation consistency significantly affected the ILI durations only in the case of long words, *t*_(159)_ = 3.66, *p* < 0.001.

**Table 3:** **Mean ILI durations (in milliseconds) and standard deviations (in parentheses) for each condition in the control and the dyslexic group**.

		**Controls**				**Dyslexics**			
		**Spelling-to-dictation**		**Direct copy transcoding**		**Spelling-to-dictation**		**Direct copy transcoding**	
		**HF**	**LF**	**HF**	**LF**	**HF**	**LF**	**HF**	**LF**
P-O consistent	Long	302 (103)	281 (100)	294 (71)	296 (65)	339 (86)	364 (150)	393 (86)	362 (72)
	Short	102 (39)	107 (40)	83 (24)	100 (41)	129 (76)	136 (73)	107 (39)	105 (43)
P-O inconsistent	Long	325 (103)	326 (73)	291 (67)	276 (79)	394 (112)	391 (99)	370 (107)	346 (102)
	Short	98 (31)	109 (38)	85 (28)	88 (33)	140 (83)	158 (118)	103 (35)	112 (41)

### Writing durations

These were defined as the time between the first pen down in a word and the last pen lift in the same word. Thus, this term refers to the duration of writing the whole word. The main effect of group, *F*_(1, 38)_ = 6.24; *p* < 0.05; *MSE* = 35,990,000; η^2^_*p*_ = 0.14, was significant. The control group (mean = 2271 ms) wrote faster than the dyslexic group (mean = 2746 ms). The main effect of type of task, *F*_(1, 38)_ = 18.48; *p* < 0.001; *MSE* = 4,852,167.31; η^2^_*p*_ = 0.33, was also significant, with longer writing durations in spelling-to-dictation (mean = 2421 ms) than in direct copy transcoding (mean = 2596). In spite of the fact that the main effects of consistency, word frequency and word length were significant (and some of their interactions) we will not comment on the results obtained for these variables in the analysis on written durations. These effects involve comparisons between different words, so they are likely to reflect differences in the duration of the hand movements required to produce different letters. An obvious example is the word length effect; such an effect in writing durations would be merely due to the fact that more letters have to be produced in the case of long words. Moreover, different letters may yield very different writing durations. For this reason, we consider that an interpretation of writing durations is recommended only when the same items are compared. Thus, we decided to comment only on the main effects of group and type of task observed for this dependent variable.

### Accuracy

Table 4 shows the mean percentage of correct responses and standard deviations in each condition for both groups. The main effects of group, *F*_(1, 38)_ = 15.31; *p* < 0.001; *MSE* = 7562.5; η^2^_*p*_ = 0.29, type of task, *F*_(1, 38)_ = 7.58; *p* < 0.01; *MSE* = 1562.5; η^2^_*p*_ = 0.17, consistency, *F*_(1, 38)_ = 11.91; *p* < 0.005; *MSE* = 1562.5; η^2^_*p*_ = 0.24, and word length, *F*_(1, 38)_ = 7.83; *p* < 0.01; *MSE* = 1128.91; η^2^_*p*_ = 0.17, were significant. The dyslexic group made more errors than the control group. More errors were made in the spelling-to-dictation than in the copying task. Inconsistent words yielded more errors than consistent words, and long words more than short words. The interaction Word length × Group was also significant, *F*_(1, 38)_ = 4.58; *p* < 0.05; *MSE* = 660.16; η^2^_*p*_ = 0.11. *T*-tests revealed that the dyslexic group made more errors in long than in short words, *t*_(159)_ = 3.01, *p* < 0.005. However, the error rates in the control group were not affected by word length, *t* < 1.

**Table 4:** **Mean percentage of correct responses and standard deviations (in parentheses) for each condition in the control and the dyslexic group**.

		**Controls**				**Dyslexics**			
		**Spelling-to-dictation**		**Direct copy transcoding**		**Spelling-to-dictation**		**Direct copy transcoding**	
		**HF**	**LF**	**HF**	**LF**	**HF**	**LF**	**HF**	**LF**
P-O consistent	Long	96.25 (2.8)	97.5 (2.66)	97.5 (1.89)	100 (2.71)	87.5 (2.8)	90 (2.66)	96.25 (1.89)	88.75 (2.71)
	Short	98.75 (2.48)	96.25 (2.36)	98.75 (1.7)	100 (1.22)	91.25 (2.48)	92.5 (2.36)	96.25 (1.7)	97.5 (1.22)
P-O inconsistent	Long	93.75 (2.97)	95 (3.78)	97.5 (2.68)	97.5 (3.88)	86.25 (2.97)	82.5 (3.78)	88.75 (2.68)	83.75 (3.88)
	Short	96.25 (3.05)	93.75 (3.48)	98.75 (2.25)	97.5 (2.92)	90 (3.05)	87.5 (3.48)	95 (2.25)	91.25 (2.92)

## General discussion

In the present study, we aimed to establish whether or not adults with dyslexia showed impaired spelling abilities, and how their performance differed from that of their peers without dyslexia. Namely, we addressed the relative contribution of the orthographic lexical information, the P-O conversion procedures and the graphemic buffer during direct copy transcoding and spelling-to-dictation in both dyslexic and normal readers. In order to evaluate the impact of these processes during each task, we manipulated the lexical frequency, the P-O consistency and the length of to-be-written Spanish words.

Our results revealed that the dyslexics produced longer written latencies, longer ILIs, longer writing durations and more errors than the controls. The dyslexic group was slower not only in preparing but also in producing the word. It is therefore confirmed that spelling difficulties in developmental dyslexia persist into adulthood. Even though all the dyslexic participants had undertaken or were undertaking at the moment higher education, the dyslexic group showed slower and poorer performance than the control group. Moreover, this group effect interacted with the type of task in the analysis of written latencies. For the control group, latencies were longer in the spelling-to-dictation task than in the direct copy transcoding task; meanwhile the dyslexic group showed the opposite pattern. However, it is important to note that the dyslexics were significantly slower than the controls in both tasks. It seems that in the dyslexic group the access to the orthographic (output) representation (as reflected by written latencies) was impaired, especially when the input was a written word. This interaction was absent in the analysis conducted on ILIs and writing durations, indicating that, once the response has been initiated, the nature of the task does not produce different effects depending on the group.

For both groups, ILIs were longer during the spelling-to-dictation task than during direct copy transcoding, and the dyslexics produced longer ILIs and writing durations than the controls. Altogether, this pattern of results might indicate that the serially-ordered production of letters is more difficult from an auditory than from a visually presented word, and that the dyslexics have a deficit affecting this process regardless of whether or not the sequence is present. This finding is line with the idea that developmental dyslexia is characterized by an impairment of serial-order learning (Szmalec et al., 2011). The existence of a difficulty in keeping the correct sequence of letters of the to-be-written word could account for the longer ILIs produced by dyslexic than by non-dyslexic participants.

Written latencies were affected by word frequency, P-O consistency and word length, while ILI durations revealed significant effects only of P-O consistency and word length. This pattern of results confirms that written latencies and ILI durations tap into different processes (Afonso, 2014). It also confirms that word frequency does not influence the course of the written response, but only written latencies. In contrast, P-O consistency did affect the duration of the critical interval, indicating that inconsistency is processed during the ILI preceding the inconsistent grapheme. Also word length influenced ILI durations, with longer ILIs being produced for long compared to short words. In both groups high-frequency, P-O consistent and short words yielded shorter written latencies than low-frequency, P-O inconsistent and long words respectively. However, significant interactions between some of these variables and the variable group reflected that some of these effects were different for the dyslexic than for the non-dyslexic participants.

Concerning word frequency, the effect observed in written latencies was larger for the dyslexic than for the control group. It seems that low-frequency words were especially problematic for the dyslexics. However, P-O consistency equally affected both groups. Thus, our results do not support the existence of differences in the application of the P-O conversion patterns between control and dyslexic adults, at least in a language with a transparent orthography such as Spanish. The dyslexic participants showed no differences compared to the controls in the size of P-O consistency. This evidence indicates that spelling deficits observed in the group with developmental dyslexia could be better accounted for by the existence of difficulties linked to lexical representations than to problems with the application of P-O conversion rules (Holmes and Castles, 2001; Di Betta and Romani, 2006).

The word length effect was larger for the dyslexic group than for the control group, but reached significance only in the analysis of the duration of the ILIs and in the analysis of errors (in written latencies this effect was marginally significant). As previously mentioned, this effect is thought to reflect the cost of additional processing loads at the graphemic buffer. The fact that the dyslexic group was more affected than the control group by the length of the stimuli in the ILI durations but not in the written latencies may suggest that the damage to the graphemic buffer associated with developmental dyslexia mainly affects the maintenance of serial-order information for subsequent production. If individuals with dyslexia have difficulties distinguishing between the constitutive letters of the stimuli (Szmalec et al., 2011; Tops et al., 2012), then this would make it difficult for them to correctly select the grapheme that has to be produced. In contrast, written latencies would be less affected by a deficit at the graphemic buffer level, since the written response can be initiated as soon as the first letters of the word are identified. This interpretation is in line with the fact that in the analysis of written latencies the word length effect was larger in direct copy transcoding than in spelling-to-dictation. It has been argued that in the spelling-to-dictation task spelling is accomplished from left to right and that it might be initiated before the whole word is processed (Bonin et al., 2001b). Thus, before the written response has been initiated, less involvement of the buffer would be required in spelling-to-dictation than in copy transcoding, in which all the letters of the word are available at the same time. In contrast, ILIs are longer in spelling-to-dictation than in direct copy transcoding in both groups, indicating that more effort is required to retrieve non-initial letters in the former than in the latter task.

Moreover, adult writers with normal reading initiated their response faster in the direct copy transcoding than in the spelling-to-dictation task. This effect reflects the fact that in the copying task the orthographic representation is present, so the appropriate word form can be directly activated in the orthographic output lexicon by the input (by means of a link between orthographic input and output representations) or by the semantic system (Cuetos, 1991). In both cases, the slower sublexical route may have no opportunity to influence the written response or the ILIs. In contrast, during spelling-to-dictation both lexical and sublexical information are activated by the auditory input (Bonin et al., 2001b; Tainturier and Rapp, 2001). Accordingly, we obtained word frequency effects in both tasks, but P-O consistency effects were observed only in the spelling-to-dictation task. This pattern of results is in line with the evidence recently obtained by Bonin et al. (2014). These authors reported shorter written latencies in immediate copying than in spelling-to-dictation, and a significant effect of P-O consistency only in spelling-to-dictation.

The significant effect of word length confirmed that orthographic representations are kept in a buffer which is sensitive to the number of elements that have to be maintained. This representation consists of a set of graphemes that have to be produced in the correct serial order. From left to right, these graphemes are identified and submitted to more peripheral processes (in the case of handwriting, retrieval of the corresponding abstract motor program, size control and/or muscular adjustment). Increased processing demands at the graphemic buffer (as exerted by long words) affected both latencies and ILI durations, indicating that the abstract representation kept in this working memory system is used throughout the whole course of the written response.

The evidence obtained in the present study indicates that these spelling problems are due to impairments at the lexical and at the graphemic buffer level. On the one hand, the dyslexic group was more affected by lexical frequency than the control group, suggesting that only high-frequency words have strong lexical representations in developmental dyslexia. The existence of failures in the orthographic lexical knowledge in dyslexia has been proposed by several authors (Goulandris and Snowling, 1991; Hanley et al., 1992; Angelelli et al., 2004; Di Betta and Romani, 2006), and our results confirm that these failures persist over time. On the other hand, the dyslexic group showed a marked word length effect compared to the control group. This effect is consistent with impairment at the graphemic buffer level. As mentioned above, a deficit in this buffer is characterized by the presence of errors such as deletions, omissions or transpositions, which are common errors in developmental dyslexia. The specific nature of the underlying deficit cannot be concluded from the evidence obtained in the present study. However, the hypothesis claiming that dyslexics have problems learning serial-order information (Szmalec et al., 2011) might account for the observed results. According to Szmalec, dyslexia may affect the Hebb repetition effect. This effect is defined as better recall for repeating than for non-repeating sequences, and it is associated with the learning of serial order information. Impairment in the Hebb repetition learning would be consistent with the presence of difficulties in keeping long sequences in the short-term memory system and for generating a long-term representation from this serially-ordered information. More evidence is necessary to determine whether or not a problem with the Hebb repetition effect could be at the basis of the spelling difficulties observed in the present study.

The dyslexic adults did not differ from the non-dyslexic group in the P-O consistency effect. Although studies conducted in other languages with transparent orthographies have reported difficulties in the application of the P-O conversion rules in dyslexic children (Caravolas and Violin, 2001; Angelelli et al., 2010), we did not find evidence of such an impairment in Spanish adults with dyslexia. These results are consistent with the claim that spelling difficulties in developmental dyslexia might vary along the lifespan (Wimmer, 1996; Bishop, 1997; Snowling and Nation, 1997; Landerl and Wimmer, 2000; Angelelli et al., 2010; Tops et al., 2012). Namely, they confirmed that problems with P-O correspondences as shown by children with dyslexia are largely remediated in adulthood. Perhaps an initial difficulty to learn the appropriate correspondences between phonemes and graphemes in childhood is overcome due to contact with the language (Di Betta and Romani, 2006). It seems reasonable to think that a sufficient knowledge of the P-O rules of a language such as Spanish, which is largely transparent concerning the phoneme-to-grapheme correspondences, may be achieved by dyslexics through years of practice in their application. This may be particularly true in our study, in which the dyslexic participants are highly educated and their frequent exposure to written material is assured.

The effect of the type of task was very different between the dyslexic and the non-dyslexic group. The dyslexics produced longer written latencies in direct copy transcoding than in spelling-to-dictation, and the controls showed the opposite pattern. However, the dyslexic group was slower than the control group in both cases. In future investigations it should be established if this difference reflects a deficiency in producing orthographic representations from visually-presented words or if it reveals separate and accumulative deficits of reading and writing in direct copy transcoding. For example, it would be interesting to test if the same pattern is observed in a delayed copy transcoding task. If similar results were obtained when participants had enough time for reading the word, the fact that the dyslexics were slower in copying than in spelling-to-dictation could not be attributed to additional problems during the reading process.

In sum, the present study provides evidence of the presence of spelling difficulties in developmental dyslexia. Even when dyslexics produced correct written responses, the writing process differed from that of normal-reading peers. More importantly, our results are consistent with deficits at the lexical level and at the graphemic buffer level. In contrast, adults with dyslexia showed no differences with the controls in the application of the P-O conversion procedures. Our results also provide valuable information about the normal writing production process, and specifically about the variations in this process due to the characteristics of the task. However, more evidence is needed to establish the scope of the involvement of lexical and sublexical information in languages with less transparent correspondences between phonemes and graphemes, and how the difference between languages might affect the spelling impairments showed by dyslexics.

### Conflict of interest statement

The authors declare that the research was conducted in the absence of any commercial or financial relationships that could be construed as a potential conflict of interest.
