# Supplementary material for: Spelling impairments in Spanish dyslexic adults
Source: Front Psychol. 2015 Apr 20;6:466. doi: 10.3389/fpsyg.2015.00466 (PMC4403306; doi:10.3389/fpsyg.2015.00466)
Supplement: Supplementary file 1 [file DataSheet1.DOCX]

**Appendix A**

Experimental stimuli used in the writing tasks.

|  | **Words** | **WF** | **NL** | **NS** | **N** | **AoA** |
| --- | --- | --- | --- | --- | --- | --- |
|  | *Consistent* |  |  |  |  |  |
|  | *High-frequency* |  |  |  |  |  |
|  | *Short* |  |  |  |  |  |
|  | Átomo | 13.57 | 5 | 3 | 2 | 7.73 |
|  | Edad | 154.46 | 4 | 2 | 0 | 5 |
|  | Pluma | 15.89 | 5 | 2 | 0 | 4.27 |
|  | Salud | 79.64 | 5 | 2 | 1 | 4.7 |
|  | *Long* |  |  |  |  |  |
|  | Escalera | 38.21 | 8 | 4 | 0 | 3.07 |
|  | Iglesia | 107.86 | 7 | 3 | 0 | 3.43 |
|  | Pistola | 26.79 | 7 | 3 | 0 | 4.87 |
|  | Teléfono | 80.18 | 8 | 4 | 1 | 7 |
|  | *Low-frequency* |  |  |  |  |  |
|  | *Short* |  |  |  |  |  |
|  | Ataúd | 5.54 | 5 | 3 | 0 | 5.43 |
|  | Dedal | 0.54 | 5 | 2 | 2 | 3.63 |
|  | Fresa | 2.86 | 5 | 2 | 2 | 4.13 |
|  | Tigre | 4.29 | 5 | 2 | 0 | 5.17 |
|  | *Long* |  |  |  |  |  |
|  | Mariposa | 6.25 | 8 | 4 | 0 | 3.43 |
|  | Palmera | 0.54 | 7 | 3 | 2 | 5.6 |
|  | Prismáticos | 4.11 | 11 | 4 | 0 | 5.9 |
|  | Trompeta | 3.04 | 8 | 3 | 0 | 5.3 |
|  | *Inconsistent* |  |  |  |  |  |
|  | *High-frequency* |  |  |  |  |  |
|  | *Short* |  |  |  |  |  |
|  | Avión | 50.36 | 5 | 2 | 1 | 4.37 |
|  | Imagen | 163.93 | 6 | 3 | 0 | 5.33 |
|  | Nube | 16.79 | 4 | 2 | 0 | 4.4 |
|  | Sabor | 24.46 | 5 | 2 | 3 | 4.13 |
|  | *Long* |  |  |  |  |  |
|  | Bicicleta | 11.96 | 9 | 4 | 0 | 4.2 |
|  | Gabinete | 13.21 | 8 | 4 | 0 | 6.33 |
|  | Televisión | 146.79 | 10 | 4 | 0 | 7.3 |
|  | Ventana | 93.93 | 7 | 3 | 2 | 3.47 |
|  | *Low-frequency* |  |  |  |  |  |
|  | *Short* |  |  |  |  |  |
|  | Abeja | 3.57 | 5 | 3 | 3 | 3.87 |
|  | Escoba | 2.86 | 6 | 3 | 0 | 3.13 |
|  | Fobia | 3.93 | 5 | 2 | 0 | 7.2 |
|  | Tabú | 3.04 | 4 | 2 | 1 | 7.1 |
|  | *Long* |  |  |  |  |  |
|  | Bolígrafo | 5.54 | 7 | 4 | 1 | 5.5 |
|  | Calabaza | 2.5 | 8 | 4 | 0 | 4.27 |
|  | Taburete | 4.46 | 8 | 4 | 0 | 5.53 |
|  | Vidriera | 1.61 | 8 | 3 | 0 | 6.37 |

*Note*. **WF** = word frequency; **NL** = number of letters; **NS** = number of syllables; **N** = orthographic neighborhood; **AoA** = age of acquisition.
